# IFN-γ production by brain-resident cells activates cerebral mRNA expression of a wide spectrum of molecules critical for both innate and T cell-mediated protective immunity to control reactivation of chronic infection with *Toxoplasma gondii*

**DOI:** 10.3389/fcimb.2023.1110508

**Published:** 2023-02-15

**Authors:** Yasuhiro Suzuki, Jenny Lutshumba, Kuey Chu Chen, Mohamed H. Abdelaziz, Qila Sa, Eri Ochiai

**Affiliations:** ^1^ Department of Microbiology, Immunology and Molecular Genetics, University of Kentucky College of Medicine, Lexington, KY, United States; ^2^ Department of Pharmacology and Nutritional Science, University of Kentucky College of Medicine, Lexington, KY, United States; ^3^ Genomics Core Laboratory, University of Kentucky College of Medicine, Lexington, KY, United States

**Keywords:** IFN-γ, protective immunity, *Toxoplasma gondii*, brain-resident cells, chemokine, cytokine, antigen presentation, regulatory molecule

## Abstract

We previously demonstrated that brain-resident cells produce IFN-γ in response to reactivation of cerebral infection with *Toxoplasma gondii*. To obtain an overall landscape view of the effects of IFN-γ from brain-resident cells on the cerebral protective immunity, in the present study we employed NanoString nCounter assay and quantified mRNA levels for 734 genes in myeloid immunity in the brains of T and B cell-deficient, bone marrow chimeric mice with and without IFN-γ production by brain-resident cells in response to reactivation of cerebral *T. gondii* infection. Our study revealed that IFN-γ produced by brain-resident cells amplified mRNA expression for the molecules to activate the protective innate immunity including 1) chemokines for recruitment of microglia and macrophages (CCL8 and CXCL12) and 2) the molecules for activating those phagocytes (IL-18, TLRs, NOD1, and CD40) for killing tachyzoites. Importantly, IFN-γ produced by brain-resident cells also upregulated cerebral expression of molecules for facilitating the protective T cell immunity, which include the molecules for 1) recruiting effector T cells (CXCL9, CXCL10, and CXCL11), 2) antigen processing (PA28αβ, LMP2, and LMP7), transporting the processed peptides (TAP1 and TAP2), assembling the transported peptides to the MHC class I molecules (Tapasin), and the MHC class I (H2-K1 and H2-D1) and Ib molecules (H2-Q1, H-2Q2, and H2-M3) for presenting antigens to activate the recruited CD8^+^ T cells, 3) MHC class II molecules (H2-Aa, H2-Ab1, H2-Eb1, H2-Ea-ps, H2-DMa, H2-Ob, and CD74) to present antigens for CD4^+^ T cell activation, 4) co-stimulatory molecules (ICOSL) for T cell activation, and 5) cytokines (IL-12, IL-15, and IL-18) facilitating IFN-γ production by NK and T cells. Notably, the present study also revealed that IFN-γ production by brain-resident cells also upregulates cerebral expressions of mRNA for the downregulatory molecules (IL-10, STAT3, SOCS1, CD274 [PD-L1], IL-27, and CD36), which can prevent overly stimulated IFN-γ-mediated pro-inflammatory responses and tissue damages. Thus, the present study uncovered the previously unrecognized the capability of IFN-γ production by brain-resident cells to upregulate expressions of a wide spectrum of molecules for coordinating both innate and T cell-mediated protective immunity with a fine-tuning regulation system to effectively control cerebral infection with *T. gondii*.

## 1 Introduction

*Toxoplasma gondii* is an obligate intracellular protozoan parasite that can establish a chronic infection in the brain (Montoya and Liesenfeld, 2004; Dubey, 2007). This chronic infection is based on the formation of tissue cysts of the parasite. This infection is prevalent in humans worldwide including developed countries (Montoya and Liesenfeld, 2004). Based on the prevalence of IgG antibodies to *T. gondii*, which is a standard method to identify the infected individuals, one third of human populations is estimated to be infected with this parasite (Montoya and Liesenfeld, 2004). This chronic infection can reactivate and cause potentially serious toxoplasmic encephalitis (TE), when infected hosts become immunocompromised due to AIDS, neoplasms, or immunosuppressive treatments for organ transplantation (Montoya and Liesenfeld, 2004). The reactivation of chronic *T. gondii* infection is initiated by ruptures of the tissue cysts, followed by release of bradyzoites from the cysts, conversion of the released bradyzoites to tachyzoites (the acute stage form of the parasite), and proliferation of the tachyzoites.

IFN-γ is required to prevent the cerebral tachyzoite proliferation and the development of TE (Suzuki et al., 1989). CD8^+^ T cells, and CD4^+^ T cells at lesser extent, are crucial producers of the IFN-γ to prevent cerebral tachyzoite growth (Gazzinelli et al., 1991; Suzuki et al., 1994; Wang et al., 2005). However, our previous studies identified that the in addition to the T cells, innate immune cells other than NK cells need to produce IFN-γ to prevent reactivation of chronic *T. gondii* infection (Kang and Suzuki, 2001). Whereas adoptive transfer of immune T cells from infected wild-type (WT) mice to infected athymic nude mice, which lack T cells but have innate immune cells capable of producing IFN-γ, is able to prevent reactivation of cerebral *T. gondii* infection in the recipients, the transfer of WT immune T cells to infected IFN-γ-knockout mice fails to prevent the reactivation of the infection (Kang and Suzuki, 2001). In addition, a depletion of NK cells from infected nude mice by treatment with anti-asialo GM1 antibody does not ablate the capability of the mice to prevent TE after receiving a transfer of immune T cells (Kang and Suzuki, 2001).

In regard to the non-NK innate immune cells that produce IFN-γ, both CD45^+^CD11b^low^ microglia and CD45^+^CD11b^high^ blood-derived macrophages purified from the brains of infected, T cell-deficient mice during reactivation of cerebral *T. gondii* infection are identified to secrete IFN-γ when they are cultured without any additional stimulations *in vitro* (Wang and Suzuki, 2007). Our recent studies with bone marrow (BM) chimeras revealed that brain-resident cells (non-hematopoietic cells in the brain), but not hematopoietic innate immune cells, need to produce IFN-γ in addition to T cells to prevent TE (Sa et al., 2015). Based on this evidence, in the present study we utilized NanoString myeloid innate immunity panel capable of measuring mRNA levels for 734 immunity-related genes to obtain a comprehensive view of the effects of IFN-γ production by brain-resident cells on the cerebral immune responses during reactivation of cerebral *T. gondii* infection using the bone marrow chimeric mice with and without IFN-γ production by brain-resident cells. We found that the IFN-γ production by brain-resident cells upregulate mRNA expressions for a wide spectrum of molecules for recruitment and activation of the cells crucial for the innate and T cell-mediated protective immunity to effectively control cerebral tachyzoite proliferation along with increasing an expression of down-regulatory molecules for preventing overly stimulated immune responses that can cause unwanted immunopathology.

## 2 Materials and methods

### 2.1 Mice

Female BALB/c, and BALB/c-background RAG1^-/-^ and IFN-γ^-/-^ mice were from the Jackson Laboratories (Bar Harbor, ME). Female Swiss-Webster mice were from Taconic (Germantown, NY). Mice were housed in specific pathogen-free environment and experimental procedures were performed in sterile settings. The studies were performed in accordance with approved protocols from the Institutional Animal Care and Use Committee. Two independent studies were performed and the results from those two studies were combined for the analyses.

### 2.2 Generation of BM chimeric mice

RAG1^-/-^ and IFN-γ^-/-^ mice received whole body irradiation (950 rads), and 1-2 hrs later, the animals were injected intravenously with 2.4 x 10^7^ BM cells from RAG1^-/-^ mice (namely RAG1^-/-^→RAG1^-/-^ and RAG1^-/-^→IFN-γ^-/-^, respectively). BM cells were prepared from tibial and femoral bones of the donor mice and suspended in Hanks’ balanced salt solution (HBSS, Hyclone, Logan, UT) with 2% fetal bovine serum (FBS, Sigma, St. Louis, MO). Animals received sulfamethoxazole/trimethoprim (0.8 mg/ml for the former and 0.16mg/ml for the latter) in drinking water beginning at 1 week before the irradiation for 5 weeks.

### 2.3 Infection with *T. gondii*

Cysts of the ME49 strain were obtained from brains of chronically infected Swiss-Webster mice (Kang and Suzuki, 2001). BM chimeric mice were infected with 10 cysts orally by gavage at two weeks after discontinuation of sulfamethoxazole/trimethoprim treatment. Animals were then treated with sulfadiazine in the drinking water (400 mg/L) beginning at 4-6 days after infection for 2-3 weeks to control the proliferation of tachyzoites and establish a chronic infection in their brains (Kang and Suzuki, 2001). Their brains were collected on the last day of sulfadiazine treatment (Day 0) and Day 5 after discontinuation of sulfadiazine, which had initiated reactivation of the cerebral infection with *T. gondii*. The Day 0 is the baseline time point without active tachyzoite proliferation in the brain, and the Day 5 is the early stage of an occurrence of the progressive active tachyzoite proliferation in the brains to elucidate the immune responses activated during the early stage of reactivation of the infection.

### 2.4 RNA extraction and NanoString gene expression analysis

RNA was isolated from a half brain of each of infected BM chimeric mice using RNA STAT-60 (Tel-test, Friendswood, TX) as previously described (Ochiai et al., 2015) at Day 0 and Day 5 after discontinuation of sulfadiazine treatment. The RNA concentration was measured using Nanodrop ND-2000 (Thermo Fisher Scientific, Waltham MA). Total RNA (100 ng) from each brain sample was quality checked using a Bioanalyzer for RNA Integrity Score (RIN> 9.0) and used to measure mRNA levels for 734 immunity-related genes using NanoString nCounter Mouse Myeloid Innate Immunity Panel (NanoString Technologies, Seattle, WA). The assay was performed by the Genomics Core Laboratory at the University of Kentucky. Data were collected and analyzed using the NanoString SPRINT nCounter and nSolvr 4.0 software (NanoString Technologies), and normalized using positive control and housekeeping genes. When transcript counts of some genes were less than the background value of the assay, which is the average of the negative control transcripts, the data on those genes were excluded from analysis. The major advantage of the use of the NanoString nCounter assay system is that we can apply 12 individual RNA samples to one assay plate for simultaneously quantifying mRNA levels for the 734 immunity-related genes. In this way, we can apply RNA samples from 2 or 3 mice on Day 0 and 3 or 4 mice on Day 5 from each of the RAG1^-/-^→RAG1^-/-^ and RAG1^-/-^→IFN-γ^-/-^ mouse groups from one experiment to a single assay plate and perform the quantification of the mRNA levels in all of the 12 samples in the identical condition. Two independent studies were performed using the BM chimeric mice, and the mRNA expression levels in the samples from each of these two experiments were measured independently using the same NanoString gene panel. Thereafter, the results from these two studies were combined for the analyses. The total numbers of the samples were five for Day 0 and seven for Day 5 for each of the two groups of BM chimeric mice.

### 2.5 Statistical analysis

Differences in mRNA expression levels of molecules in NanoString data between experimental groups were determined by ANOVA using PartekFlow software (Partek Inc. MO). Differences that provided *P*<0.05 were considered significant.

## 3 Results and discussion

### 3.1 Expression of mRNA for the molecules involved in the IFN-γ-mediated signaling pathway is upregulated only in the presence of brain-resident cells capable of producing IFN-γ in response to reactivation of cerebral infection with *T. gondii*

We recently developed an experimental model to examine the roles of IFN-γ production by brain-resident cells in prevention of reactivation of cerebral *T. gondii* infection (Sa et al., 2015). In this model, RAG1^-/-^ or IFN-γ^-/-^ mice were treated with a whole body irradiation to eradicate hematopoietic cells and thereafter, they received a systemic transfer of BM cells obtained from RAG1^-/-^ mice that lack T and B cells but have innate immune cells such as NK cells and macrophages that can produce IFN-γ (namely RAG1^-/-^→RAG1^-/-^ and RAG1^-/-^→IFN-γ^-/-^, respectively) (Sa et al., 2015). The brain-resident cells including microglia are irradiation-resistant. Therefore, the only difference between the brains of these two groups of BM chimeric mice was the presence (RAG1^-/-^→RAG1^-/-^ mice) and the absence (RAG1^-/-^→IFN-γ^-/-^ mice) of the capability to produce IFN-γ in the brain-resident cells under the presence of the hematopoietic innate immune cells capable of producing this cytokine in the systemic circulation (Sa et al., 2015). Our previous study using this model demonstrated that RAG1^-/-^→RAG1^-/-^ mice inhibited reactivation of cerebral *T. gondii* infection approximately 10 times more effectively than did RAG1^-/-^→IFN-γ^-/-^ mice, and that the efficient control of the reactivation of the infection in the former was associated with approximately 18 times increase in cerebral IFN-γ protein levels, which occurred only in RAG1^-/-^→RAG1^-/-^ mice, during reactivation of the infection (Sa et al., 2015). In the present study, we utilized this BM chimeric mice model and the NanoString nCounter assay system to obtain an overall landscape view of the effects of IFN-γ production by brain-resident cells on the cerebral immune responses.

**Figure 1:**
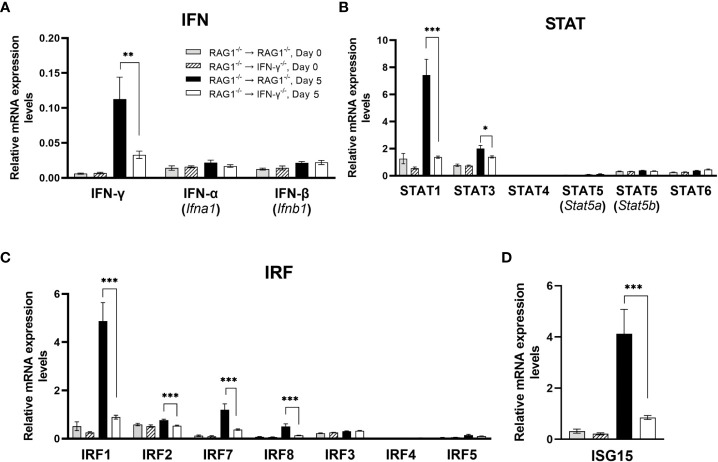
Cerebral mRNA levels for IFN-γ-mediated signaling pathway are upregulated only in the presence of brain-resident cells capable of producing IFN-γ in response to reactivation of cerebral infection with *T. gondii.* RAG1^-/-^→RAG1^-/-^ and RAG1^-/-^→IFN-γ^-/-^ mice were infected orally with 10 cysts of the ME49 strain of *T. gondii* and treated with sulfadiazine in the drinking water (400 mg/L) beginning at 4-6 days after infection for 2-3 weeks to control the proliferation of tachyzoites and establish a chronic infection in their brains. Their brains were collected on the last day of sulfadiazine treatment (Day 0) and Day 5 after discontinuation of sulfadiazine (Day 5), which had initiated reactivation of the cerebral infection with *T. gondii*. The mRNA levels for **(A)** type I IFN (IFN-α and IFN-β) and type II IFN (IFN-γ), **(B)** STAT1, 3, 4, 5 and 6, **(C)** IRF1, 2, 3, 4, 5, 7, and 8, and **(D)** ISG15 in the brains of both groups of mice at both Day 0 and Day 5 were measured using NanoString nCounter Mouse Myeloid Innate Immunity Panel. *P<0.05, **P<0.01, and ***P<0.001. The data were combined from two independent experiments (n=5 for Day 0 and n=7 for Day 5 in both RAG1^-/-^→RAG1^-/-^ and RAG1^-/-^→IFN-γ^-/-^ mice).

RAG1^-/-^→RAG1^-/-^ than RAG1^-/-^→IFN-γ^-/-^ mice were infected with *T. gondii* and treated with sulfadiazine to control tachyzoite proliferation and establish a chronic infection in their brains. Three to four weeks after the infection, sulfadiazine treatment was discontinued to initiate reactivation of cerebral *T. gondii* infection. Brains samples were collected from these mice on the last day of sulfadiazine treatment (Day 0) and 5 days after its discontinuation (Day 5). The mRNA levels for the immunity-related genes on Day 0 indicate the baseline expression levels for these genes during the latent chronic *T. gondii* infection without active tachyzoite proliferation in their brains. The mRNA levels on Day 5 indicate their expression levels in response to an occurrence of reactivation of the infection during the early stage of the progressive active tachyzoite proliferation in the brains of these two groups of mice. The mRNA levels for IFN-γ in response to reactivation of cerebral *T. gondii* infection (Day 5) were markedly higher in the brains of RAG1^-/-^→RAG1^-/-^ than RAG1^-/-^→IFN-γ^-/-^ mice as expected (*P*<0.01), whereas the mRNA levels for this cytokine at Day 0 were similarly low in both of these two groups of animals (**Figure 1A**). In contrast, mRNA levels for both IFN-α (*Ifna1*) IFN-β (*Ifnb1*) did not differ between the brains of these two groups of mice (**Figure 1A**). Consistently, mRNA levels for the key molecules involved in IFN-γ signaling, signal transducer and activator or transcription 1 (STAT1) and IFN regulatory factor 1 (IRF1), were both more than 5 times grater in the brains of RAG1^-/-^→RAG1^-/-^ than RAG1^-/-^→IFN-γ^-/-^ mice (*P*<0.001 for the both) (**Figures 1B, C**). The mRNA levels for three other IRF molecules that are involved in the IFN-γ signaling (Elser et al., 2002; Kuwata et al., 2002; Farlik et al., 2012) were also significantly upregulated in the former than the latter (1.4-fold increase for IRF2, 3.2-fold increase for IRF7, and 3.8-fold increase for IRF8, *P*<0.001 for all of these molecules, **Figure 1C**). In contrast, mRNA levels for the other STAT (STAT 4, 5a, 5b, and 6) and IRF molecules (IRF3, 4, and 5) did not differ between these two groups (**Figures 1B, C**). Although type I interferon can also upregulate IRF2, IRF7, and IRF8 expression, mRNA levels for both IFN-α and IFN-β were not upregulated in the brains of RAG1^-/-^→RAG1^-/-^ mice when compare to those of RAG1^-/-^→IFN-γ^-/-^ mice as described earlier, suggesting that the upregulation of mRNA for these three IRF molecules are due to the increased IFN-γ expression in the former. These results indicate that IFN-γ production by brain-resident cells in response to reactivation of cerebral *T. gondii* infection is effective to potently activate the IFN-γ signaling pathway to activate the protective immunity to suppress tachyzoite growth and reactivation of the infection.

Of interest, mRNA levels for interferon-stimulated gene 15 (ISG15) was also 4.9 times greater in the brains of RAG1^-/-^→RAG1^-/-^ than RAG1^-/-^→IFN-γ^-/-^ mice (*P*<0.001) (**Figure 1D**). A recent study showed that ISG15^-/-^ mice have reduced IFN-γ levels in their sera when compared to WT control mice during the acute stage of *T. gondii* infection (Napolitano et al., 2018). Therefore, it may be possible that the increased expression of ISG15 contribute to amplifying IFN-γ production by brain-resident cells. ISG15 has also been shown to be involved in killing of tachyzoites within IFN-γ-activated human (HeLa) cells (Bhushan et al., 2020).

### 3.2 IFN-γ production by brain-resident cells amplifies mRNA expression for the molecules to activate the innate immune functions in response to reactivation of cerebral *T. gondii* infection

#### 3.2.1 Molecules involved in recruitment of microglia, monocytes, and macrophages

**Figure 2:**
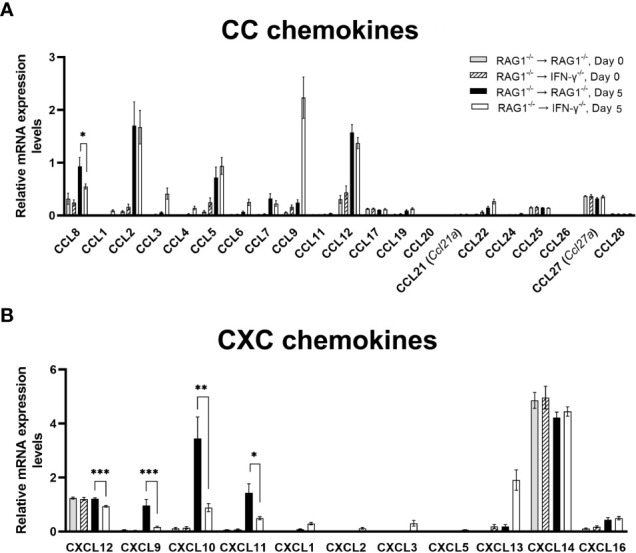
Cerebral mRNA levels for the molecules that recruit microglia, monocytes, macrophages, and T cells are upregulated by IFN-γ production by brain-resident cells during reactivation of cerebral *T. gondii* infection. For the details of experimental methods, see the legend of **Figure 1**. The mRNA levels for **(A)** 21 CC chemokines and **(B)** 11 CXC chemokines were measured at both Day 0 and Day 5 in the brains of both groups of mice were measured using NanoString nCounter Mouse Myeloid Innate Immunity Panel. *P<0.05, **P<0.01, and ***P<0.001. The data were combined from two independent experiments (n=5 for Day 0 and n=7 for Day 5 in both RAG1^-/-^→RAG1^-/-^ and RAG1^-/-^→IFN-γ^-/-^ mice).

Recent studies by others demonstrated an importance of Ly6C^+^ inflammatory monocytes in controlling tachyzoite growth in the brains of mice (Biswas et al., 2015). Among 21 CC chemokines that we tested, mRNA expression levels for only CCL8 were 1.7 times greater in the brains of RAG1^-/-^→RAG1^-/-^ than RAG1^-/-^→IFN-γ^-/-^ mice (*P*<0.05) (**Figure 2A**). CCL8 is a chemokine that can recruit various types of immune cell populations including monocytes and macrophages (Charo and Ransohoff, 2006). Although we were unable to find the information on the role of CCL8 in the resistance against *T. gondii*, it has been reported that CCL8 is associated with control of cytomegalovirus replication in organ transplant recipients (Lisboa et al., 2015).

In regard to CXC chemokines, mRNA levels for CXCL12 along with CXCL9, CXCL10, and CXCL11, were significantly greater in the brains of RAG1^-/-^→RAG1^-/-^ than RAG1^-/-^→IFN-γ^-/-^ mice at Day 5 after initiation of reactivation of cerebral *T. gondii* infection (*P*<0.001) (**Figure 2B**). CXCL12 is able to stimulate transmigration of monocytes across the blood-brain barrier *in vitro* (Man et al., 2012). A unique characteristic of this chemokine is that both glial cells and neurons can produce this chemokine (Guyon, 2014). A recent study using a human neuroblastoma cell line demonstrated that transfection of this cell line to express *T. gondii* rhoptry protein 16 (ROP16) induces increased expression of CXCL12 (Fan et al., 2016). ROP16 is one of the molecules that *T. gondii* secretes when it invade into host cells. Therefore, it is most likely that neurons, possibly glial cells as well, produce CXCL12 when they become infected with *T. gondii* tachyzoites. Another study in humans also showed that increased serum levels of CXCL12 are a marker for an occurrence of ocular toxoplasmosis (Marino et al., 2020). Therefore, it is possible that greater levels of CXCL12 mRNA in the brains of RAG1^-/-^→RAG1^-/-^ mice contribute to increased recruitment of inflammatory monocytes for efficient control of cerebral tachyzoite growth during reactivation of *T. gondii* infection, when compared to infected RAG1^-/-^→IFN-γ^-/-^ mice.

CXCR3 is the receptor for each of CXCL9, CXCL10, and CXCL11, and this chemokine receptor is expressed on microglia, resident macrophages in the brain parenchyma, in both normal and disease conditions (Rappert et al., 2004; de Haas et al., 2008; Krauthausen et al., 2014). Microglia activated by IFN-γ are able to kill tachyzoites *in vitro* (Chao et al., 1993). Whereas the direct evidence on an expression of CXCR3 on microglia in the brain during *T. gondii* infection is not available yet, our recent studies demonstrated that CXCR3 expression significantly increases during CD8^+^ T cell-mediated anti-*T. gondii* cyst immune responses (Lutshumba et al., 2020), in which Iba1^+^ microglia and Ly6C^+^ inflammatory macrophages accumulate to the cysts and phagocytose bradyzoites within the cysts for their elimination (Tiwari et al., 2019). Therefore, it is possible that CXCR3^+^ microglia accumulate into the areas of tachyzoite proliferation and phagocytose them to kill the parasite.

#### 3.2.2 Molecules involved in increasing phagocytosis of microglia and macrophages

**Figure 3:**
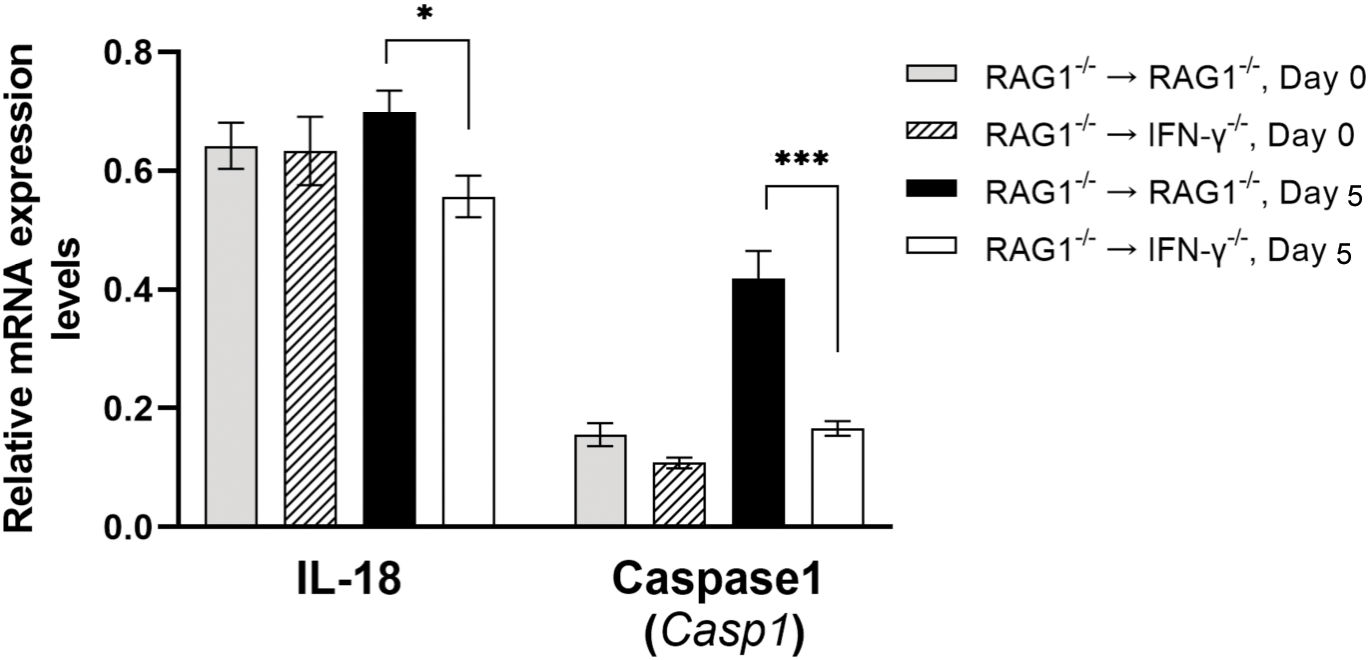
Cerebral mRNA levels for molecules that increase phagocytosis of microglia and macrophages are upregulated by IFN-γ production by brain-resident cells during reactivation of cerebral *T. gondii* infection. For the details of experimental methods, see the legend of **Figure 1**. The mRNA levels for IL-18 and caspase1, which generate active IL-18 molecule from pro-IL-18, were measured at both Day 0 and Day 5 in the brains of both groups of mice were measured using NanoString nCounter Mouse Myeloid Innate Immunity Panel. *P<0.05 and ***P<0.001. The data were combined from two independent experiments (n=5 for Day 0 and n=7 for Day 5 in both RAG1^-/-^→RAG1^-/-^ and RAG1^-/-^→IFN-γ^-/-^ mice).

IL-18 can enhance phagocytosis of microglia and macrophages (Mori et al., 2001; Henan et al., 2014). Of interest, mRNA expression levels for IL-18 were significantly greater in the brains of RAG1^-/-^→RAG1^-/-^ than RAG1^-/-^→IFN-γ^-/-^ mice during reactivation of cerebral *T. gondii* infection (*P*<0.05) (**Figure 3**). Caspase-1 cleaves the precursor of IL-18 and activates this cytokine. In agreement, expression levels of mRNA for caspase-1 were 2.5 times greater in the brains of RAG1^-/-^→RAG1^-/-^ than RAG1^-/-^→IFN-γ^-/-^ mice (*P*<0.001) (**Figure 3**). An importance of IL-18 in resistance against *T. gondii* was recently demonstrated by using an acute infection model in mice deficient in IL-18 and IL-18 receptor (IL-18R) (Gorfu et al., 2014). They demonstrated that wild-type mice had markedly increased IL-18 levels in sera during the acute stage of infection, and that both IL-18^-/-^ and IL-18R^-/-^ mice showed increased mortality associated with enhanced parasite proliferation during the infection (Gorfu et al., 2014). Consistently, a recent study showed that mice deficient in caspase-1/11 display decreased serum IL-18 levels and increased parasite replication and mortality (Gorfu et al., 2014). Another recent study using macrophages from *T. gondii*-resistant LEW rats demonstrated that pharmacological inactivation of caspase-1 ablated the capability of their macrophages to kill tachyzoites *in vitro* (Cavailles et al., 2014), although it is not addressed whether this anti-tachyzoite activity of caspase-1 is mediated by IL-18 or not.

#### 3.2.3 Molecules involved in recognition of the pathogen-associated molecular patterns including Toll-like receptors, and NOD-like receptors

**Figure 4:**
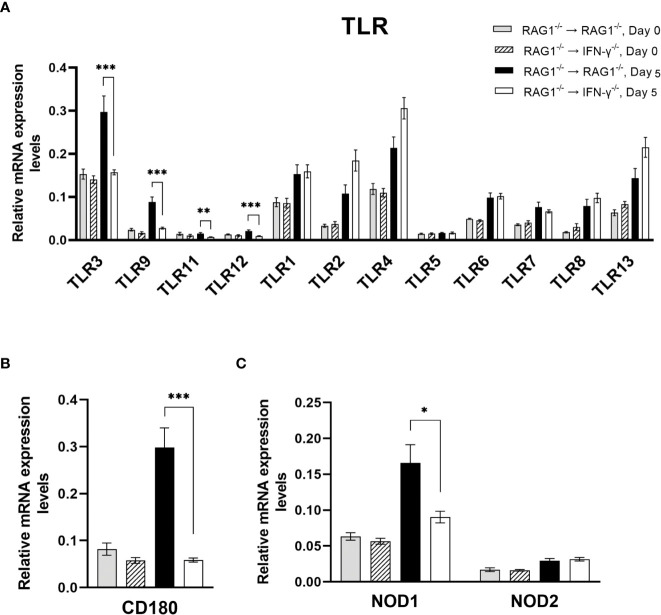
Cerebral mRNA levels for the molecules that recognize the pathogen-associated molecular patterns are upregulated by IFN-γ production by brain-resident cells during reactivation of cerebral *T. gondii* infection. For the details of experimental methods, see the legend of **Figure 1**. The mRNA levels for **(A)** 12 TLRs, **(B)** CD180 (a member of TLR family), and **(C)** NOD1 and NOD2 were measured at both Day 0 and Day 5 in the brains of both groups of mice were measured using NanoString nCounter Mouse Myeloid Innate Immunity Panel. *P<0.05, **P<0.01, and ***P<0.001. The data were combined from two independent experiments (n=5 for Day 0 and n=7 for Day 5 in both RAG1^-/-^→RAG1^-/-^ and RAG1^-/-^→IFN-γ^-/-^ mice).

Among 11 TLR tested, mRNA levels for TLR3, TLR9, TLR11, and TLR12 were approximately 2-3 times greater in the brains of RAG1^-/-^→RAG1^-/-^ than RAG1^-/-^→IFN-γ^-/-^ mice during reactivation of cerebral *T. gondii* infection (*P*<0.001 for TLR3, TLR9, and TLR12 and *P*<0.01 for TLR11) (**Figure 4A**). TLR11 is the first TLR that was identified to recognize a *T. gondii* molecule, profilin (Yarovinsky et al., 2005). TLR11 forms heterodimers with TLR12 to recognize *T. gondii* profilin for activating the IL-12 production (Koblansky et al., 2013; Raetz et al., 2013) and this activity is mediated by IRF8 (Raetz et al., 2013). Notably, IRF8 is one of the molecules whose mRNA levels were markedly greater in the brains of RAG1^-/-^→RAG1^-/-^ than RAG1^-/-^→IFN-γ^-/-^ mice as described earlier in section 1. TLR9 is known to recognize unmethylated CpG motifs of bacterial DNA. A previous study using WT and TLR9^-/-^ mice demonstrated that TLR9^-/-^ mice have higher parasite burdens than WT mice in the intestine during acute stage of oral infection with *T. gondii* in association with reduced frequencies of IFN-γ-producing T cells in lamina propria in the former (Minns et al., 2006). TLR3 is known to recognize double stranded RNA. A recent study demonstrated that mice deficient in TLR3/7/9 are more susceptible to *T. gondii* infection than mice deficient in TLR7/9 (Andrade et al., 2013), suggesting an involvement of TLR3 in resistance against *T. gondii* infection. Therefore, IFN-γ production by brain-resident cells in RAG1^-/-^→RAG1^-/-^ mice upregulates mRNA expressions of the TLR molecules that recognize nucleic acids and protein (profilin) of *T. gondii* to most likely amplify the protective immunity against the parasite.

Expression levels of mRNA for CD180 were 5 times greater in the brains of RAG1^-/-^→RAG1^-/-^ than RAG1^-/-^→IFN-γ^-/-^ mice during reactivation of cerebral *T. gondii* infection (*P*<0.001) (**Figure 4B**). CD180 is a member of TLR family and known as a member of microglia sensomes (Hickman et al., 2013; Saddala et al., 2021). Although the ligand for this TLR family molecule remains unknown, this sensome is induced by IFN-γ and TNF-α in microglia (Saddala et al., 2021). Therefore, it is possible that CD180 is involved in recognition of *T. gondii* tachyzoites by microglia to activate innate immunity.

Expression levels of mRNA for NOD-like receptor 1 (NOD1) were also significantly greater in the brains of RAG1^-/-^→RAG1^-/-^ than RAG1^-/-^→IFN-γ^-/-^ mice following reactivation of *T. gondii* infection (*P*<0.05) (**Figure 4C**) whereas mRNA levels for NOD2 did not differ between these two groups of mice (**Figure 4C**). Although the protective roles of NOD1 in *T. gondii* infection remain to be identified, it has been shown that NOD1 plays important roles in resistance against *Listeria monocytogenes* (Mosa et al., 2009). NOD1^-/-^ mice showed increased dissemination of the bacteria into the brains (Mosa et al., 2009). They also showed that non-hematopoietic cells mediate the NOD1-dependent resistance, and that astrocytes and fibroblasts of NOD1^-/-^ mice displayed enhanced intracellular growth of *L. monocytogenes* in *in vitro* (Mosa et al., 2009). Therefore, it may be possible that increased NOD1 mRNA levels in the brains of RAG1^-/-^→RAG1^-/-^ mice contribute to controlling intracellular proliferation of *T. gondii* tachyzoites within brain-resident cells during reactivation of the infection.

#### 3.2.4 Molecules involved in CD40

**Figure 5:**
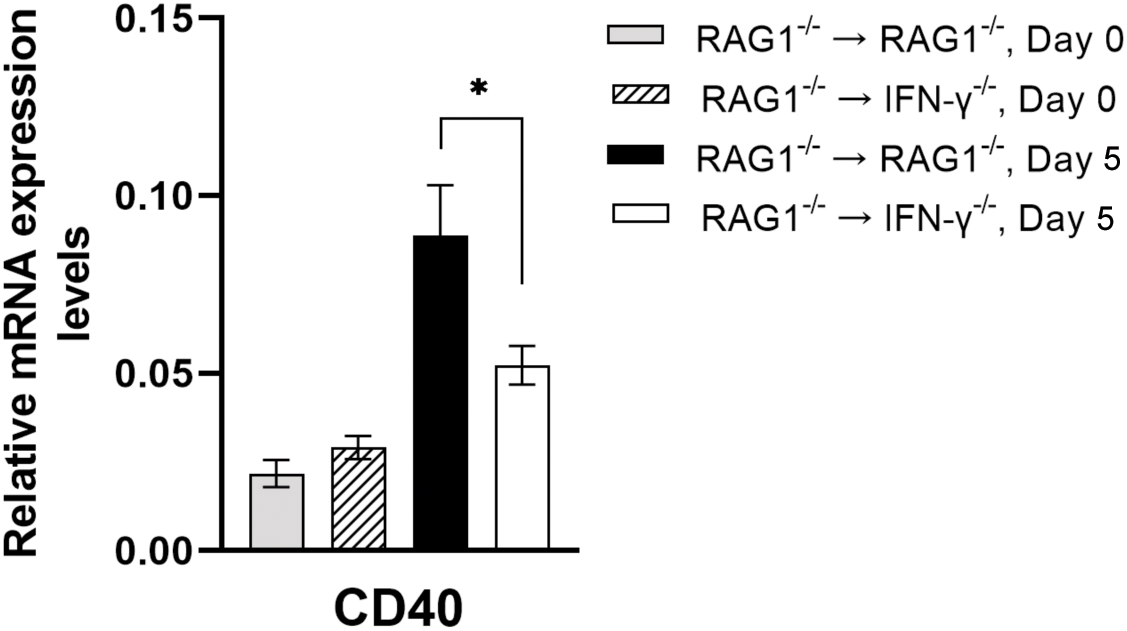
Cerebral mRNA levels for CD40, which activates intracellular killing of tachyzoites by microglia and macrophages, are upregulated by IFN-γ production by brain-resident cells during reactivation of cerebral *T. gondii* infection. For the details of experimental methods, see the legend of **Figure 1**. The mRNA levels for CD40 were measured at both Day 0 and Day 5 in the brains of both groups of mice were measured using NanoString nCounter Mouse Myeloid Innate Immunity Panel. *P<0.05. The data were combined from two independent experiments (n=5 for Day 0 and n=7 for Day 5 in both RAG1^-/-^→RAG1^-/-^ and RAG1^-/-^→IFN-γ^-/-^ mice).

Recent studies demonstrated that an activation of microglia and macrophages through CD40 expressed on their surfaces triggers killing of intracellular *T. gondii* tachyzoites (Andrade et al., 2006; Portillo et al., 2010), and that CD40^-/-^ mice are susceptible to cerebral toxoplasmosis with impaired killing of the parasite (Portillo et al., 2010). In the present study, mRNA levels for CD40 were approximately 2-fold greater in the brains of RAG1^-/-^→RAG1^-/-^ than RAG1^-/-^→IFN-γ^-/-^ mice during reactivation of *T. gondii* infection (*P*<0.05) (**Figure 5**). Astrocytes (Calingasan et al., 2002; Ke et al., 2005) and neurons (McWilliams et al., 2015; Carriba and Davies, 2021) have been shown to express CD40 ligand (CD40L). Therefore, interactions of CD40^+^ microglia and macrophages with CD40L^+^ astrocytes and/or neurons will be able to activate them to kill tachyzoites in the brain.

### 3.3 IFN-γ production by brain-resident cells amplifies mRNA expression for the molecules to facilitate the protective T cell immunity in response to reactivation of cerebral *T. gondii* infection

#### 3.3.1 Molecules involved in recruitment of CD4^+^ and CD8^+^ effector T cells

Our previous study demonstrated that the majority of both CD4^+^ and CD8^+^ T cell populations that have migrated into the brains of infected BALB/c mice are expressing CXCR3 on their cell surfaces (Ochiai et al., 2015). CXCL9, CXCL10, and CXCL11 are the three ligands for CXCR3 as mentioned earlier in section 2.1, and the expression levels for mRNA for each of these three chemokines were much greater in the brains of RAG1^-/-^→RAG1^-/-^ than RAG1^-/-^→IFN-γ^-/-^ mice (5.9 times for CXCL9 [*P*<0.001], 4.4 times for CXCL10 [*P*<0.01], and 2.9 times for CXCL11 [*P*<0.05]) (**Figure 2B**). These results on CXCL9 and CXCL10 are consistent with those that we recently reported (Sa et al., 2015). In regard to CXCL11, our previous study showed that mRNA levels for this chemokine were greater in the brains of infected WT control mice than IFN-γ^-/-^ mice, indicating that cerebral expression of this chemokine is mediated by IFN-γ during *T. gondii* infection (Wen et al., 2010).

In regard to the protective functions of these three chemokines to prevent cerebral tachyzoites growth and reactivation of *T. gondii* infection, our recent study demonstrated that CXCL9 is critical for recruiting both CD4^+^ and CD8^+^ immune T cells not only into the brain but also into the areas of tachyzoite proliferation in the brain to prevent the parasite growth in BALB/c mice that are genetically resistant to this infection (Ochiai et al., 2015). The RAG1^-/-^ and IFN-γ^-/-^ mice that were used to generate the BM chimeric mice in the present study were BALB/c-background. In regard to the roles of CXCL10, it was shown that CXCL10 is important for recruitment of CD8^+^ T cells into the brain and inhibiting tachyzoite proliferation during the progressive TE in genetically susceptible C57BL/6 mice (Harris et al., 2012). A previous study demonstrated that CXCL9 is mostly expressed in microglia, whereas CXCL10 is mostly expressed in astrocytes in the brains of infected BALB/c mice (Strack et al., 2002). Therefore, it would be possible that IFN-γ production by brain-resident cells in response to cerebral tachyzoite proliferation activates expression of CXCL9 in microglia and CXCL10 in astrocytes in the areas of tachyzoite growth and these chemokines produced by the different glial cell populations both contribute to recruiting immune T cells into those areas to prevent the pathogen growth. Although we were unable to find the information on the cell populations that express CXCL11 or the protective role of this chemokine in controlling *T. gondii* in the brain, an expression of CXCL11 in neurons has been reported in other disease models (Guo et al., 2017; Yu et al., 2019). Therefore, it would be possible that neurons in the areas of tachyzoite growth produce CXCL11 and contribute to recruiting immune T cells into those areas.

#### 3.3.2 Molecules involved in processing, transporting, and presentation of antigens through the MHC class I molecules for activating CD8^+^ T cells

CD8^+^ T cells play a crucial role in controlling cerebral tachyzoite growth to prevent reactivation of *T. gondii* infection (Gazzinelli et al., 1991; Suzuki et al., 1994; Wang et al., 2005). After CD8^+^ immune T cells migrate into the areas of tachyzoite proliferation in the brain, they need to be activated by recognizing their target antigens presented by the MHC class I molecules. This antigen-presentation process consists of the following four steps: 1) antigen processing and peptide generation, 2) peptide transport, 3) assembly of peptide-MHC class I complex, 4) presentation of the antigen by the MHC class I molecules on the surface of the cell membrane.

##### 3.3.2.1 Antigen processing and peptide generation

**Figure 6:**
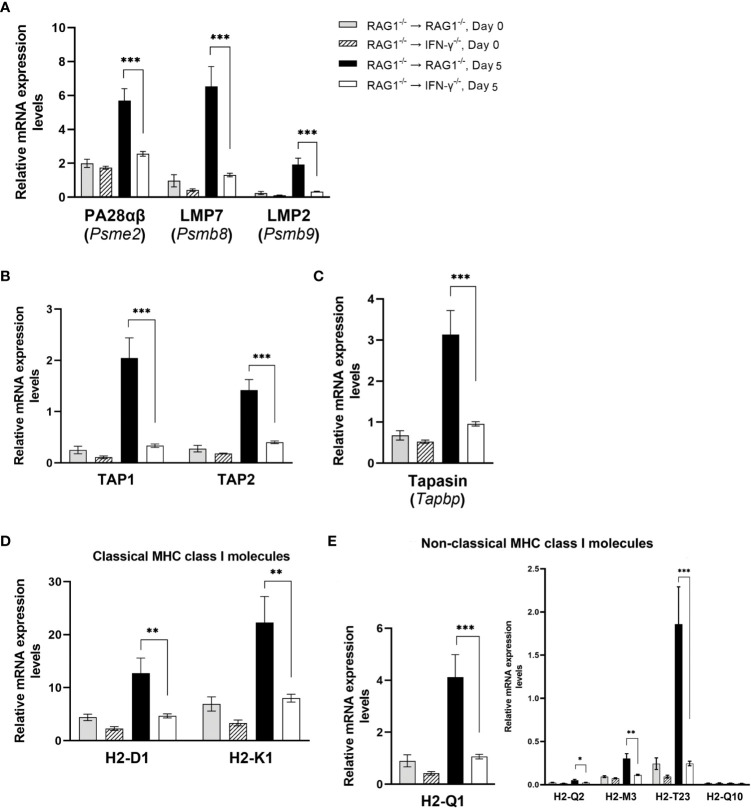
Cerebral mRNA levels for the molecule involved in processing, transporting, and presentation of antigens through the MHC class I molecules for activating CD8^+^ T cells are upregulated by IFN-γ production by brain-resident cells during reactivation of cerebral *T. gondii* infection. For the details of experimental methods, see the legend of **Figure 1**. The mRNA levels for the molecules for **(A)** antigen processing and peptide generation, **(B)** Peptide transport, **(C)** assembling peptide-MHC class I complex, and **(D**, **E)** presenting the peptides by classical MHC class I molecules **(D)** and non-classical MHC class I molecules **(E)** to activate CD8^+^ T cells were measured at both Day 0 and Day 5 in the brains of both groups of mice were measured using NanoString nCounter Mouse Myeloid Innate Immunity Panel. *P<0.05, **P<0.01, and ***P<0.001. The data were combined from two independent experiments (n=5 for Day 0 and n=7 for Day 5 in both RAG1^-/-^→RAG1^-/-^ and RAG1^-/-^→IFN-γ^-/-^ mice).

Immunoproteasome brakes ubiquitinated protein antigens into peptides and release them into cytoplasm to initiate the antigen presentation process. NanoString nCounter Mouse Myeloid Innate Immunity Panel contains three genes, *Psme2, Psmb8*, and *Psmb9*, for the molecules that compose immunoproteasome, and mRNA levels for each of these three genes were markedly greater in the brains of RAG1^-/-^→RAG1^-/-^ than RAG1^-/-^→IFN-γ^-/-^ mice (2.2 times for *Psme2*, 5.0 times for *Psmb8*, and 5.9 times for *Psmb9*, *P*<0.001 for each molecule) during reactivation of *T. gondii* infection (**Figure 6A**). Immunoproteasome gate is normally closed, and PA28αβ coded by the *Pmse2* gene is one of the regulatory particles that open the gate of the immunoproteasome (Whitby et al., 2000). The *Psmb8*, and *Psmb9* genes encode proteasome subunit-β5i (also called low molecular weight protein [LMP] 7) and proteasome subunit-β1i (also called LMP2) of immunoproteasome, respectively. These subunits are critical for generating epitopes of multiple antigens for MHC class I molecules (Sijts et al., 2000; Kincaid et al., 2011). Notably, each of these three subunits is induced by IFN-γ. Therefore, the greater mRNA levels for these three subunits of immunoproteasome in the brains of infected RAG1^-/-^→RAG1^-/-^ than RAG1^-/-^→IFN-γ^-/-^ mice are consistent with the higher IFN-γ levels derived from the production of this cytokine by brain-resident cells in the former.

##### 3.3.2.2 Peptide transport

The peptides generated by the immunoproteasone are actively transported from the cytoplasm to endoplasmic reticulum (ER) by the transporter associated with antigen processing (TAP) composed of TAP1 and TAP2, that forms a transmembrame pore in the ER membrane. Expression levels for mRNA for TAP1 and TAP2 were 6.1 and 3.5 times greater in the brains of RAG1^-/-^→RAG1^-/-^ than RAG1^-/-^→IFN-γ^-/-^ mice in response to reactivation of *T. gondii* infection (*P*<0.001 for both) (**Figure 6B**).

##### 3.3.2.3 Assembly of peptide-MHC class I complex

There are four chaperone proteins that facilitate loading of transported peptides onto the MHC class I molecules within the ER. Tapasin is one of the chaperone proteins that bridge the MHC class I molecule to TAP and allow peptides, which are transported into the ER, to gain access to the MHC class I molecules (Sadasivan et al., 1996; Lehner et al., 1998). The mRNA levels for *Tapbp*, which encodes Tapasin, in the brains of infected RAG1^-/-^→RAG1^-/-^ mice were 3.3 times greater than those of infected RAG1^-/-^→IFN-γ^-/-^ mice during reactivation of the infection (*P*<0.001) (**Figure 6C**).

##### 3.3.2.4 Antigen presentation

The assembled peptide-MHC class I complex are then placed into vesicles, and the vesicles leave the ER, pass over the Golgi, and transferred to the cell membranes to fuse with it. This process allows the peptides bound to the MHC class I molecules exposed extracellularly for activating CD8^+^ T cells. Expression levels for mRNA for classical MHC class I molecule genes, *H2-K1* and *H2-D1*, were both approximately three times greater in the brains of RAG1^-/-^→RAG1^-/-^ than RAG1^-/-^→IFN-γ^-/-^ mice during reactivation of *T. gondii* infection (*P*<0.01) (**Figure 6D**). The mRNA levels for *H2-T23*, which is the molecule predicted to be a part of the classical MHC class I protein and non-classical MHC class Ib protein complex, were 7.6 times greater in the former than the latter (*P*<0.001) (**Figure 6E**). The mRNA levels for three non-classical MHC class Ib complex molecules, H2-Q1, H-2Q2, and H2-M3, were also 2 to 3.9 times greater in the former than the latter (*P*<0.001 for *H2-Q1 P*<0.05 for *H2-Q2, P*<0.01 for *H2-M3*) (**Figure 6E**). Whereas the contributions of the non-classical MHC class Ib molecules in activating the protective CD8^+^ T cells against *T. gondii* remain to be determined, an importance of this CD8^+^ T cell activation pathway has been shown in infections with various pathogens including bacteria (D'Orazio et al., 2003) and viruses (Mazzarino et al., 2005; Swanson et al., 2008).

The non-classical MHC class Ib molecules are involved in activation of not only CD8^+^ T cells but also NK cells and NKT cells (Gomes et al., 2007). NK cells produce IFN-γ following *T. gondii* infection and contribute to controlling tachyzoite growth (Hunter et al., 1994). Therefore, the increased expression of H2-Q1, H-2Q2, H2-M3, and H2-T23 induced by IFN-γ production by brain-resident cells in RAG1^-/-^→RAG1^-/-^ mice could contribute to preventing cerebral tachyzoite proliferation by activating not only CD8^+^ T cells but also NK cells during reactivation of cerebral *T. gondii* infection. The results shown in **Figures 6A–E** all together strongly suggest that IFN-γ production by brain-resident cells in the brains of RAG1^-/-^→RAG1^-/-^ mice is able to upregulate cerebral mRNA expression for the molecules critical for all of the four steps to present *T. gondii* antigens through the MHC class I molecules to activate CD8^+^ immune T cells and possibly NK cells recruited into the brain during reactivation of cerebral *T. gondii* infection.

#### 3.3.3 Molecules involved in antigen presentation for activating CD4^+^ T cells

**Figure 7:**
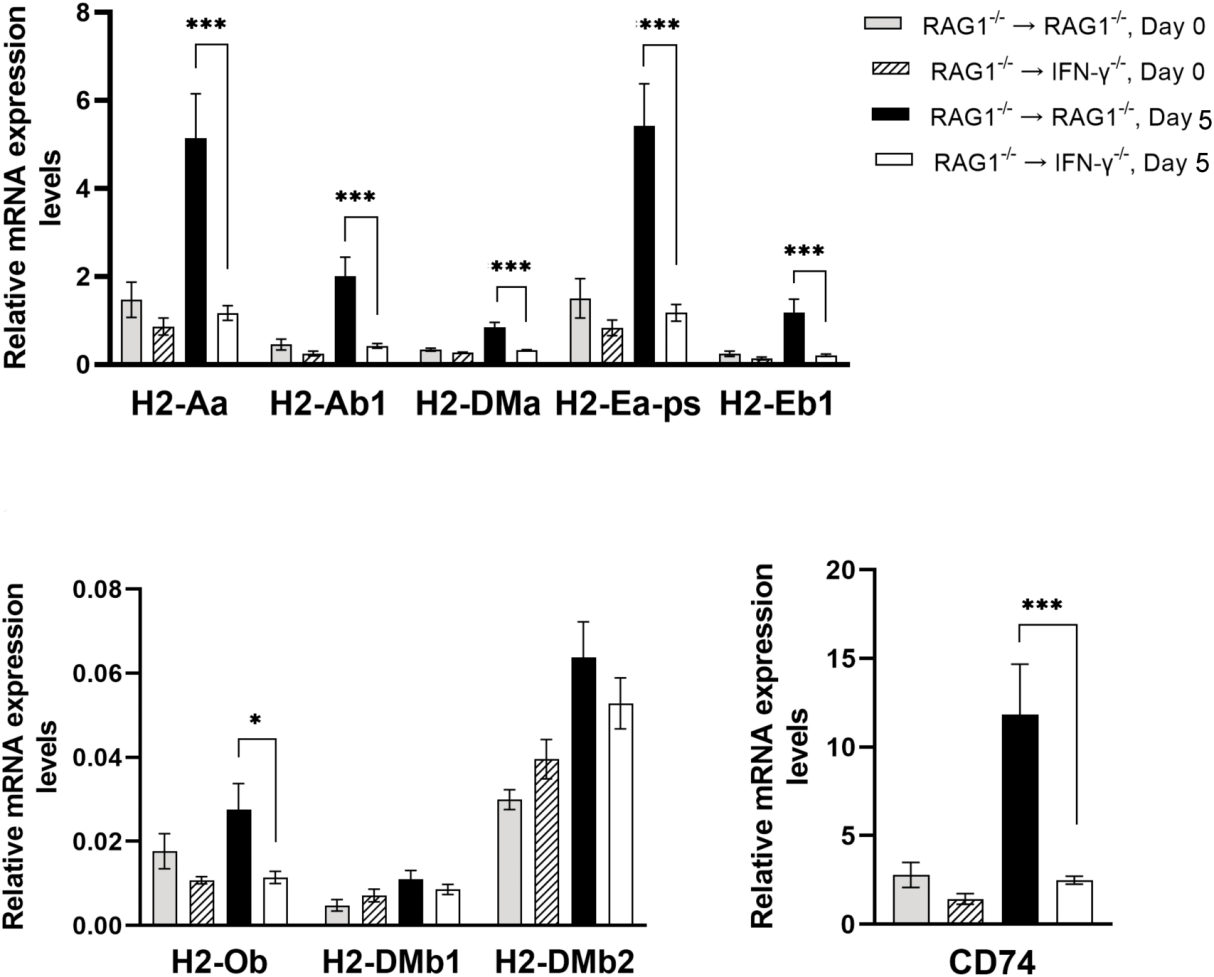
Cerebral mRNA levels for the molecules involved in antigen presentation for activating CD4^+^ T cells are upregulated by IFN-γ production by brain-resident cells during reactivation of cerebral *T. gondii* infection. For the details of experimental methods, see the legend of **Figure 1**. The mRNA levels for 9 MHC class II molecules were measured at both Day 0 and Day 5 in the brains of both groups of mice were measured using NanoString nCounter Mouse Myeloid Innate Immunity Panel. *P<0.05 and ***P<0.001. The data were combined from two independent experiments (n=5 for Day 0 and n=7 for Day 5 in both RAG1^-/-^→RAG1^-/-^ and RAG1^-/-^→IFN-γ^-/-^ mice).

CD4^+^ T cells recognize their target antigens presented by the MHC class II molecules. Among 10 MHC class II molecules tested, mRNA levels for 7 of those molecules were significantly greater in the brains of RAG1^-/-^→RAG1^-/-^ than RAG1^-/-^→IFN-γ^-/-^ mice during reactivation of *T. gondii* infection (*P*<0.001 for *H2-Aa, H2-Ab1,H2-DMa, H2-Ea-ps*, *H2-Eb1*, and CD74, and *P*<0.05 for *H2-Ob*) (**Figure 7**). These results indicate that IFN-γ production by brain-resident cells, which occurs in the brains of RAG1^-/-^→RAG1^-/-^ mice in response to reactivation of *T. gondii* infection, is able to enhance expression of most of the MHC class II molecules. In addition to CD8^+^ T cells, CD4^+^ T cells contribute to the protective immunity (Gazzinelli et al., 1991; Suzuki et al., 1994; Wang et al., 2005). Therefore, the upregulation of cerebral expression of the MHC class II molecules induced by brain-resident cell IFN-γ production most likely contribute to preventing cerebral tachyzoite growth by facilitating activation of CD4+ T cells in response to reactivation of cerebral *T. gondii* infection.

#### 3.3.4 Co-stimulatory molecules for T cell activation

**Figure 8:**
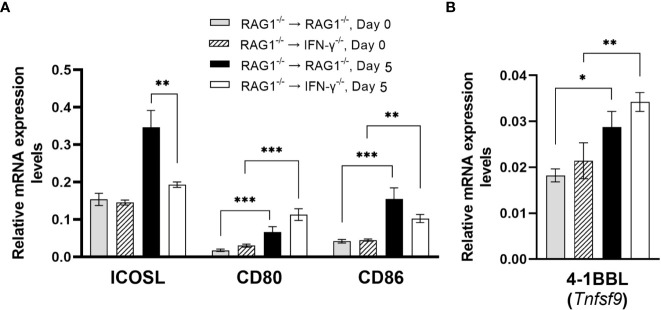
Cerebral mRNA levels for co-stimulatory molecules are upregulated by IFN-γ production by brain-resident cells during reactivation of cerebral *T. gondii* infection. For the details of experimental methods, see the legend of **Figure 1**. The mRNA levels for **(A)** ICOS, CD80, CD86 and **(B)** 4-1BBL were measured at both Day 0 and Day 5 in the brains of both groups of mice were measured using NanoString nCounter Mouse Myeloid Innate Immunity Panel. *P<0.05, **P<0.01, and ***P<0.001. The data were combined from two independent experiments (n=5 for Day 0 and n=7 for Day 5 in both RAG1^-/-^→RAG1^-/-^ and RAG1^-/-^→IFN-γ^-/-^ mice).

Interactions of CD28 expressed on CD4^+^ T cells with CD80 and CD86 expressed on antigen-presenting cells provide the co-stimulatory signal required for an activation of the T cells in addition to their recognition of target antigens presented by the MHC class II molecules. An interaction of the inducible co-stimulatory molecule (ICOS) on CD4^+^ T cells with ICOS ligand (ICOSL) on antigen-presenting cells is another co-stimulatory pathway for CD4^+^ T cells. Expression levels of mRNA for ICOSL in the brains of RAG1^-/-^→RAG1^-/-^ mice were approximately twice greater than those in RAG1^-/-^→IFN-γ^-/-^ mice during reactivation of *T. gondii* infection (*P*<0.01) (**Figure 8A**). A recent study demonstrated that ICOS is required for optimal proliferation of CD4^+^ T cells following *T. gondii* infection (Wilson et al., 2006). The absence of ICOS resulted in reduced numbers of CD4^+^ T cells and reduced production of IFN-γ by mononuclear cells isolated from the brains of infected mice following stimulation with tachyzoite lysate antigens *in vitro* (Wilson et al., 2006). Therefore, it is possible that the increased expression levels of ICOSL induced by IFN-γ production by brain-resident cells in the brains of RAG1^-/-^→RAG1^-/-^ mice contribute to enhancing proliferation and IFN-γ production of CD4^+^ T cells following their migration into their brains for efficient control of reactivation of cerebral *T. gondii* infection.

Although mRNA levels for both CD80 and CD86 did not differ between the brains of infected RAG1^-/-^→RAG1^-/-^ and RAG1^-/-^→IFN-γ^-/-^ mice during reactivation of cerebral *T. gondii* infection (**Figure 8A**), mRNA expression levels for these co-stimulatory molecules were both significantly greater after initiation of reactivation of the infection (Day 5) than their expression levels before reactivation of the infection (Day 0) in either of these two groups of BM chimeric mice (*P*<0.01or *P*<0.001) (**Figure 8A**). Previous studies showed that both CD80 and CD86 are important for proliferation and IFN-γ production of primed T cells from *T. gondii*-seropositive individuals during their secondary response to the parasite antigens *in vitro* (Subauste et al., 1998). Another study demonstrated that *T. gondii* infection triggers upregulation of CD86 in macrophages from BALB/c mice genetically resistant to cerebral *T. gondii* infection, and that the upregulated CD86 facilitate antigen-specific proliferation of CD4^+^ T cells (Fischer et al., 1999). Interestingly, the upregulation of CD86 did not occur in infected macrophages of BALB.B mice that are genetically susceptible to the infection (Fischer et al., 1999).

In activation of CD8^+^ T cells, the interaction between 4-1BB expressed on the T cells and 4-1BBL (*Tnfsf9*) expressed on the antigen-presenting cells provides the co-stimulation signal for their activation. Although mRNA levels for the *Tnfsf9* did not differ between the brains of RAG1^-/-^→RAG1^-/-^ and RAG1^-/-^→IFN-γ^-/-^ mice after reactivation of cerebral *T. gondii* infection (**Figure 8B**), their 4-1BBL mRNA levels after reactivation of the infection were significantly greater those of prior to initiation of the reactivation of the infection (*P*<0.05 or *P*<0.01) (**Figure 8B**). In our previous study, CD8^+^ T cells that migrated into the brains of RAG1^-/-^→RAG1^-/-^ mice following adoptive T cell transfer become activated and efficiently produce IFN-γ (Sa et al., 2015). Therefore, those upregulated 4-1BBL expression induced independently from IFN-γ produced by brain-resident cells in response to reactivation of *T. gondii* infection appears to provide the sufficient co-stimulatory signal for the recruited CD8^+^ T cells. As mentioned earlier, enhanced recruitment of CD8^+^ T cells and amplified antigen presentation by the MHC class I molecules induced by IFN-γ produced by brain-resident cells in RAG1^-/-^→RAG1^-/-^ mice in combination with sufficient levels of co-stimulation signal from the 4-1BB/4-1BBL interactions can most likely provide the sufficient protective immunity to control reactivation of cerebral *T. gondii* infection.

#### 3.3.5 Cytokines for facilitating IFN-γ production by NK cells and T cells

**Figure 9:**
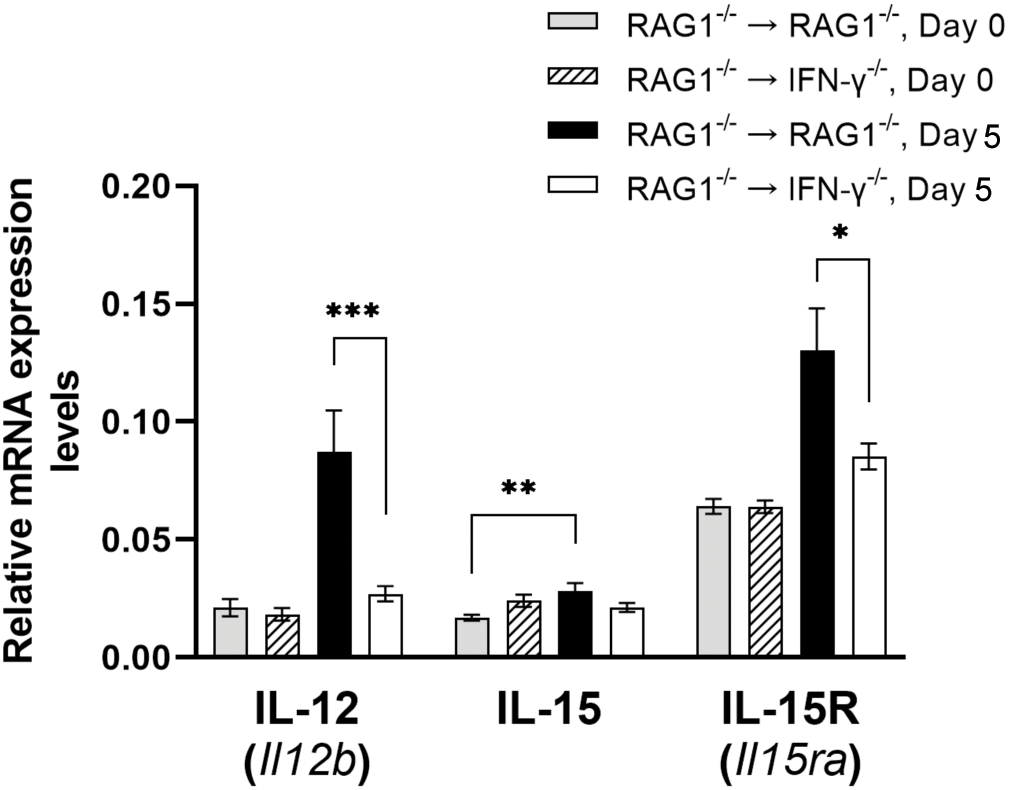
Cerebral mRNA levels for the cytokines that facilitate IFN-γ production by NK and T cells are upregulated by IFN-γ production by brain-resident cells during reactivation of cerebral *T. gondii* infection. For the details of experimental methods, see the legend of **Figure 1**. The mRNA levels for IL-12, IL-15, and IL-15R were measured at both Day 0 and Day 5 in the brains of both groups of mice were measured using NanoString nCounter Mouse Myeloid Innate Immunity Panel. *P<0.05, **P<0.01, and ***P<0.001. The data were combined from two independent experiments (n=5 for Day 0 and n=7 for Day 5 in both RAG1^-/-^→RAG1^-/-^ and RAG1^-/-^→IFN-γ^-/-^ mice).

IL-12 plays an important role in activating IFN-γ production by NK cells and T cells during *T. gondii* infection (Hunter et al., 1994). Expression levels for IL12B mRNA were 3.2 times greater in the brains of RAG1^-/-^→RAG1^-/-^ than RAG1^-/-^→IFN-γ^-/-^ mice during reactivation of cerebral *T. gondii* infection (*P*<0.001) (**Figure 9**). Therefore, IFN-γ produced by brain-resident cells in response to reactivation of the infection probably induces an environment to further facilitate IFN-γ-production by NK and T cells to prevent cerebral tachyzoite growth.

IL-15 is another cytokine that promotes the protective CD8^+^ T cell responses including both IFN-γ production and cytotoxic activity against *T. gondii* (Khan and Kasper, 1996; Bhadra et al., 2010). Whereas IL-15 mRNA levels tended to be slightly greater in the brains of RAG1^-/-^→RAG1^-/-^ than RAG1^-/-^→IFN-γ^-/-^ mice during reactivation of cerebral *T. gondii* infection, the difference did not reach statistical significance (**Figure 9**). However, when compared between before and after initiation of reactivation of the infection, only RAG1^-/-^→RAG1^-/-^ mice showed significant increases in IL-15 mRNA levels in response to reactivation of the infection (*P*<0.05) (**Figure 9**). In addition, mRNA levels for IL-15 receptor (IL-15RA) were significantly greater in the brains of the RAG1^-/-^→RAG1^-/-^ than RAG1^-/-^→IFN-γ^-/-^ mice during reactivation of *T. gondii* infection (*P*<0.05, **Figure 9**). Since IL-15 is also able to activate IFN-γ production of NK cells, the increased IL-15RA mRNA expression may reflect the activation of NK cells.

In addition to IL-12 and IL-15, mRNA expression levels for IL-18 were significantly greater in the brains of RAG1^-/-^→RAG1^-/-^ than RAG1^-/-^→IFN-γ^-/-^ mice during reactivation of cerebral *T. gondii* infection as mentioned earlier in section 3.2, (*P*<0.05, **Figure 3**). IL-18 has the capability to upregulate IFN-γ production by CD4^+^ T cells (Okamura et al., 1995; Robinson et al., 1997). A recent study using *T. gondii*-induced intestinal inflammation demonstrated that IL-18^-/-^ mice have decreased IFN-γ levels in the small intestine and serum, which are associated with decreased intestinal necrosis, following oral infection with *T. gondii* (Vossenkamper et al., 2004). We previously demonstrated that the intestinal necrosis induced by oral infection with this parasite is caused by IFN-γ production by CD4^+^ T cells (Liesenfeld et al., 1996). IL-18 also acts with IL-12 and induces IFN-γ production by CD8^+^ T cells (Okamoto et al., 1999). As described earlier in this section, IL-12 expression is markedly greater in brains of RAG1^-/-^→RAG1^-/-^ than RAG1^-/-^→IFN-γ^-/-^ mice (*P*<0.001) (**Figure 9**). Therefore, it is possible that increased expression of IL-18 induced by IFN-γ production by brain-resident cells in infected RAG1^-/-^→RAG1^-/-^ mice contributes to facilitate IFN-γ production of both CD4^+^ and CD8^+^ T cells once they are recruited into the brain in response to reactivation of cerebral *T. gondii* infection.

### 3.4 The activations of the protective innate and T cell-mediated protective immune responses induced by IFN-γ production by brain-resident cells are associated with increased mRNA expression for the molecules that down-regulate the activated protective immunity

**Figure 10:**
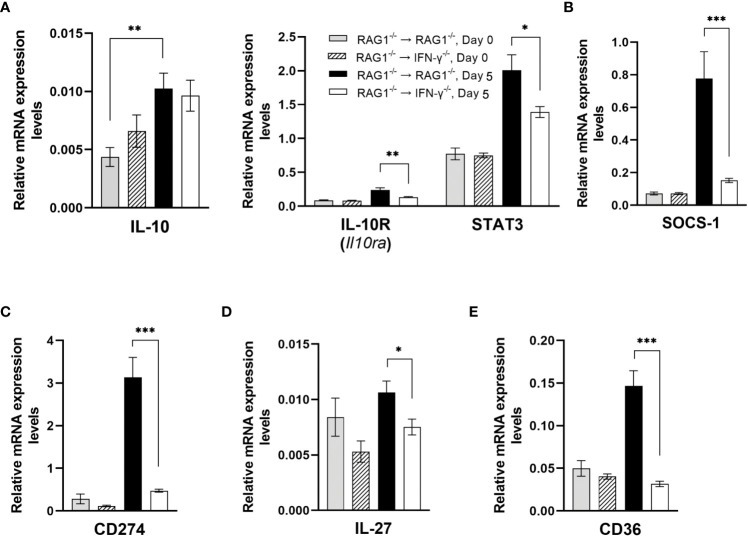
Cerebral mRNA levels for the molecules that down-regulate the IFN-γ-mediated protective immunity to prevent overly stimulated immune responses are upregulated by IFN-γ production by brain-resident cells during reactivation of cerebral *T. gondii* infection. For the details of experimental methods, see the legend of **Figure 1**. The mRNA levels for **(A)** IL-10, IL-10R, STAT3, **(B)** SOCS-1, **(C)** CD274, **(D)** IL-27, and **(E)** CD36 were measured at both Day 0 and Day 5 in the brains of both groups of mice were measured using NanoString nCounter Mouse Myeloid Innate Immunity Panel. *P<0.05, **P<0.01, and ***P<0.001. The data were combined from two independent experiments (n=5 for Day 0 and n=7 for Day 5 in both RAG1^-/-^→RAG1^-/-^ and RAG1^-/-^→IFN-γ^-/-^ mice).

Since IFN-γ is a potent proinflammatory cytokine, overly stimulated IFN-γ-mediated immune responses can cause serious tissue damages during *T. gondii* infection (Gazzinelli et al., 1996; Liesenfeld et al., 1996). Therefore, it is critical to have a finely tuned regulation of this powerful immune responses. IL-10 has been shown to play a critical role in downregulating IFN-γ-mediated protective immune responses against *T. gondii* for maintaining the protective immune responses within the desirable levels (Gazzinelli et al., 1996; Liesenfeld et al., 1996). Importantly, the potent upregulations for mRNA expression for the molecules involved in the IFN-γ-mediated protective immunity in the brains of RAG1^-/-^→RAG1^-/-^ mice were associated with relatively less (1.8-fold) but statistically significant upregulation of mRNA for IL-10R (*Il10ra*) (*P*<0.01) (**Figure 10A**). In addition, IL-10 mRNA levels significantly increased only in the brains of after the RAG1^-/-^→RAG1^-/-^ mice after reactivation of cerebral *T. gondii* infection when compared to those before the reactivation of the infection (*P*<0.05) (**Figure 10A**). The combination of increased expression levels for IL-10 and IL-10R most likely contributes to down-regulating the IFN-γ-mediated immune responses for its finely tuned regulation. This possibility is supported by the evidence that mRNA levels for the IL-10 signaling molecule, STAT3, were 1.4 times greater in the brains of RAG1^-/-^→RAG1^-/-^ than RAG1^-/-^→IFN-γ^-/-^ mice (*P*<0.05) (**Figure 10A**).

In addition to IL-10, IL-10R and STAT3, mRNA for suppressor of cytokine signaling-1 (SOCS1) was more than 5 times greater in the brains of RAG1^-/-^→RAG1^-/-^ than RAG1^-/-^→IFN-γ^-/-^ mice during the reactivation of cerebral *T. gondii* infection (*P*<0.001) (**Figure 10B**). SOCS1 can inhibit STAT1-mediaed signaling for IFN-γ by directly inhibiting JAK1/2 (Liau et al., 2018). Therefore, an activation of IFN-γ production by brain-resident cells in RAG1^-/-^→RAG1^-/-^ mice in response to reactivation of the infection occurs in association with upregulations of multiple downregulatory mechanisms to secure an optimum protection against cerebral tachyzoite proliferation without causing unwanted tissue damages caused by overly stimulated IFN-γ-mediated inflammatory responses.

An interaction between CD274 (PD-L1) and CD279 (PD-1) is a key component of the immune checkpoint inhibitors that prevent unwanted overly stimulated or overly prolonged immune responses. Expression levels of mRNA for CD274 (PD-L1) were also more than 6 times greater in the brains of RAG1^-/-^→RAG1^-/-^ than RAG1^-/-^→IFN-γ^-/-^ mice (*P*<0.001) (**Figure 10C**).

IL-27 is also an important downregulator of the Th1 CD4^+^ T cell responses. Expression levels of IL-27 mRNA were significantly greater in the brains of RAG1^-/-^→RAG1^-/-^ than RAG1^-/-^→IFN-γ^-/-^ mice in response to reactivation of *T. gondii* infection (*P*<0.05) (**Figure 10D**). It has been shown that IL-27 limits IL-2 production by the Th1 cells to prevent their overly stimulated IL-2 production that can cause a lethal inflammatory immunopathology associated with highly upregualted Th1 immune responses during acute *T. gondii* infection (Villarino et al., 2006). IL-27 also downregulates the development of Th17 cells during *T. gondii* infection, and IL-27R^-/-^ mice developed severe inflammatory histopathology associated a prominent IL-17 response in the brain during the later stage of the infection and showed increased mortality (Stumhofer et al., 2006).

Expression levels of mRNA for CD36 (scavenger receptor) markedly increased in the brains of RAG1^-/-^→RAG1^-/-^ mice when compared to RAG1^-/-^→IFN-γ^-/-^ mice during reactivation of *T. gondii* infection (*P*<0.001) (**Figure 10E**). A recent study demonstrated that CD36^-/-^ mice displayed increased weight losses and mortality associated with heightened IFN-γ response by NK cells as well as increased serum levels for biomarkers for tissue damages, GDF-15 and FGF-12 (Zhao et al., 2021). Therefore, the increased CD36 expression induced by IFN-γ production by brain-resident cells in the brains of RAG1^-/-^→RAG1^-/-^ mice could be involved in the finely tuned regulation of the IFN-γ-mediated protective immunity in the brain during reactivation of cerebral *T. gondii* infection to prevent tissue damages.

The only difference in the brains between RAG1^-/-^→RAG1^-/-^and RAG1^-/-^→IFN-γ^-/-^ mice is the presence (the former) and the absence (the latter) of the capability to produce IFN-γ in the brain-resident cells in the presence of the hematopoietic innate immune cells capable of producing this cytokine in the systemic circulation (Sa et al., 2015) as mentioned earlier. The present study revealed that the IFN-γ production by brain-resident cells in *T. gondii*-infected RAG1^-/-^→RAG1^-/-^ mice significantly upregulates cerebral expressions of mRNA for a wide spectrum of molecules critical for both innate and T cell-mediated protective immunity to control tachyzoite growth during reactivation of chronic infection. These molecules include the molecules involved in IFN-γ signaling pathway, confirming that IFN-γ production by brain-resident cells is indeed able to activate the signaling pathway of this cytokine against cerebral tachyzoite proliferation. In the protective innate immunity, brain-resident cell IFN-γ production enhanced the expression of molecules for chemokines for recruitment of microglia and macrophages (CXCL9, CXCL10, and CXCL11) and an activation of those recruited phagocytes (IL-18, TLR family molecules [TLR3, TLR9, TLR11, TLR12, and CD180], NOD1, and CD40) for killing tachyzoites during reactivation of cerebral infection with *T. gondii*.

For activation of T cell-mediated protective immunity, the present study revealed that the IFN-γ production by brain-resident cells in *T. gondii*-infected RAG1^-/-^→RAG1^-/-^ mice is able to upregulate cerebral expressions of mRNA for chemokines for recruiting CD4^+^ and CD8^+^ effector T cells into the areas of tachyzoite proliferation (CXCL9, CXCL10, and CXCL11) and the molecules for presenting target antigens through the MHC class I and II molecules to activate the recruited CD4^+^ and CD8^+^ T cells. The upregulated molecules for activating CD8^+^ T cells includes those for the processing of antigens (PA28αβ, LMP2, and LMP7), transporting the processed peptides (TAP1 and TAP2), assembling the transported peptides to the MHC class I molecules (Tapasin), and the MHC class I (H2-K1 and H2-D1) and MHC class Ib molecules (H2-Q1, H-2Q2, H2-M3, and H2-T23). For CD4^+^ T cell activation, the present study showed upregulations of mRNA for eight MHC class II molecules (H2-Aa, H2-Ab1, H2-DMa, H2-Ea-ps, H2-Eb1, H2-Ob, and CD74) through IFN-γ production by brain-resident cells. In addition, our study revealed that brain-resident cell-derived IFN-γ is able to upregulate cerebral expression of co-stimulatory molecules (ICOSL), and cytokines (IL-12, IL-15, and IL-18) facilitating IFN-γ production by NK cells and CD4^+^ and CD8^+^ T cells.

Since IFN-γ-mediated protective immunity is a powerful proinflammatory process, an important aspect from the host defense is to maintain this protective immunity within desirable levels through finely tuned regulatory mechanisms to avoid overly stimulated or overly prolonged IFN-γ-mediated pro-inflammatory responses that cause serious tissue damages. Notably, the present study revealed that IFN-γ production by brain-resident cells in *T. gondii*-infected RAG1^-/-^→RAG1^-/-^ mice also upregulates cerebral expressions of mRNA for those down-regulatory molecules (IL-10, IL-10R, STAT3, SOCS1, CD274 [PD-L1], IL-27, and CD36) in response to reactivation of the infection to maintain the protective immune responses within a desirable range for an effective host defense. Thus, the present study uncovered the previously unrecognized but highly effective capability of IFN-γ production by brain-resident cells to orchestrate both the innate and T cell-mediated protective immunity with a fine-tuning regulation system to control cerebral infection with *T. gondii*. IFN-γ plays crucial roles in resistance against infections with various intracellular microorganisms in the brain (Chesler and Reiss, 2002; van den Broek et al., 1995). Therefore, the finely regulated activation of the innate and T cell-mediated protective immunity organized by IFN-γ production by brain-resident cells presented in the present study using *T. gondii* infection could function as a key first line defense system in the brain to effectively control cerebral infections with the other various microorganisms as well. Performing further functional analyses will be able to elucidate the mechanisms by which the IFN-γ production by brain-resident cells effectively orchestrate cerebral protective immune responses against the infection.

## Data availability statement

The original contributions presented in the study are included in the article/supplementary material. Further inquiries can be directed to the corresponding author.

## Ethics statement

The animal study was reviewed and approved by IACUC committee at the University of Kentucky.

## Author contributions

Designing research studies: YS. Funding acquisition: YS. Conducting experiments and acquiring the data: YS, JL, QS, and EO. Analyzing the data: KC, MA, and YS. Writing manuscript: YS, Reviewing the manuscript: YS, JL, KC, MA, QS, and EO. All authors contributed to the article and approved the submitted version.
